# Chronology of gastrointestinal cancer

**DOI:** 10.1007/s00595-017-1574-y

**Published:** 2017-08-09

**Authors:** Kentaro Murakami, Hisahiro Matsubara

**Affiliations:** Department of Frontier Surgery, Graduate School of Medicine, Chiba University, 1-8-1 Inohana, Chuo-ku, Chiba, 260-8670 Japan

**Keywords:** Chronology, Gastrointestinal cancer, Tumor growth rate

## Abstract

The “chronology of cancer” is a concept that describes the nature of cancers through the measure of time. The field extends from carcinogenesis to development, progression, and metastasis. Carcinogenesis is a multi-step process, which results from the accumulation of multiple genetic or epigenetic alterations. Various chronologies of gastrointestinal cancers have been reported for carcinogenesis caused by different risk factors. These chronologies are useful for developing cancer prevention strategies. The tumor growth rate is one of the most important factors in this field. Combining the factors of time and tumor growth enables us to estimate the time at which cancer or metastasis occurred, retrospectively, and to predict the survival of cancer patients, prospectively. It is noteworthy that these chronologies differ significantly among individual cases, even of cancers derived from the same organ. Thus, they are useful for individualization. We can apply the knowledge obtained in this field to the basic research and the diagnosis and treatment of cancers. The chronology of cancer is a classical but interesting field, which helps us consider and explore the essence of cancer. We review the topics related to the chronology of gastrointestinal cancer, ranging from carcinogenesis to metastasis.

## Introduction

Kusama proposed the concept of the “chronology of cancer” to describe the nature of cancers through the measure of time [1]. Observation of the various phenomena that occur in a cancer patient over time reveals aspects of the cancer, ranging from carcinogenesis to development, progression, and metastasis [2–10]. The knowledge obtained from this field can be applied to the diagnosis, treatment, and basic research of cancer. Thus, the concept of chronology is essential to the optimal surveillance interval of endoscopy [11], the period or interval of postoperative follow-up [12], and the treatment efficacy, with a focus on survival [13]. The chronology of cancer is a classical field, which has existed, since the mid-20th century. Recently, many studies on the malignancy of cancers have focused on the genome sequence [14–16], and findings from these studies suggest in correlation with the tumor growth rate. Furthermore, the multi-step carcinogenesis process, consisting of the initiation, promotion, and progression of the cancer, has also been studied in genetic or epigenetic fields [17–19]. By adding the concept of chronology to these findings, we can consider the carcinogenic mechanism sterically. From this perspective, the concept of the chronology of cancer is still important. Therefore, it can be said that the chronology of cancer is an old but new field. We performed a literature search of two electronic bibliographic databases (PubMed and Igaku Chuo Zassi) to identify studies published before October, 2016, on the chronology of gastrointestinal cancer.

## Carcinogenesis

One of the most powerful initiators of cancer is tobacco smoke, which contains mutagens, such as benzopyrene and dimethylnitrosamine. The most well-known gastrointestinal cancer related to smoking is esophageal cancer. A large-scale cohort study of approximately 110,000 people in Japan demonstrated an increasing risk of esophageal carcinogenesis with heavier smoking. The hazard ratio of death caused by esophageal cancer was 2.05 in the <25.0 smoking-years group, 3.54 in the 25.1–35.0 smoking-years group, 5.34 in the 35.1–45.0 smoking-years group, and 4.85 in the >45.1 smoking-years group, compared with non-smokers [2]. In the Netherlands cohort study, the duration of cigarette smoking was 36.9 ± 10.5 years in patients with esophageal squamous cell carcinoma and 34.0 ± 11.0 years in patients with esophageal adenocarcinoma [3]. Thus, the duration from the first exposure of carcinogens to the formation of cancer is a few decades. Achalasia and Barrett’s esophagus have also been identified as risk factors for esophageal cancer. Achalasia is a rare motility disorder of the esophagus, in which increased bacterial growth and chemical irritation from the continuous decomposition of food and saliva can induce malignant transformation of esophageal epithelial cells [4]. The mean duration from the onset of symptoms caused by achalasia until the detection of esophageal cancer was reported to be 24.9 years [5]. Barrett’s esophagus, defined as intestinal metaplasia in the distal esophagus, is considered a complication of gastroesophageal reflux disease and a precursor lesion in most cases of esophageal adenocarcinoma [20]. Recent progress in molecular biology has implicated several genetic and epigenetic alterations in both the carcinogenesis and progression of esophageal cancer [21–24]. The progression to cancer in seven patients with Barrett’s esophagus was marked by low-grade dysplasia and then high-grade dysplasia. The main outcome measure was the time from the first diagnosis of intestinal metaplasia. Low-grade dysplasia developed after a median of 24 months, high-grade dysplasia after a median of 33 months, and cancer after a median of 36 months [6]. Chronic *Helicobacter pylori* gastritis has been described as an important risk factor for the development of atrophic gastritis, intestinal metaplasia, and gastric cancer. One study compared the ratio of the degree of intestinal metaplasia and the histological types (differentiated or undifferentiated) according to age groups. The curves for both were parallel. The study found that severe intestinal metaplasia and differentiated-type gastric cancer increased with the age of the patients. The distance between the two curves was approximately 10 years and the author speculated that it took 10 years for gastric cancer to develop through atrophic gastritis and intestinal metaplasia [7]. Colorectal tumorigenesis has also been regarded as a multi-step process related to the accumulation of genetic alterations with two potential oncogenic pathways: the adenoma–carcinoma sequence pathway and de novo carcinogenesis. While the latter has persisted strongly in Japan, at present, it is believed that the former is the main route to the development of colorectal cancers [25]. It generally takes 5–15 years for a small adenoma to develop into a malignancy [8, 9]. Furthermore, patients with long-standing ulcerative colitis have an increased risk of the development of colorectal cancer. A previous study on the clinicopathological features of 312 patients with ulcerative colitis and colorectal cancer demonstrated the mean disease duration from the onset of ulcerative colitis to the detection of cancer to be 14.2 years. The number of cases of an elapse of more than 7 years was 253 (85%) and that of more than 10 years was 219 (73%) [10].

## Tumor growth rate

The major axis of a single cell is approximately 10 μm. The diameter of tumors consisting of 10^9^ cells is 1 cm, which is the size of detection by medical imaging modalities such as X-rays and CT scans. Tumors consisting of 10^12^ cells are approximately 10 cm in diameter, which often results in tumor metastasis and potentially patient death.

Most cancers are derived from a single abnormal cell that has undergone transformation [26]. A single cell divides to make two cells by one round of cell division. If all the tumor cells divide repeatedly and do not die, a single cell grows into two cells, two into four, four into eight, and so on. In other words, cells grow exponentially to 2^*n*^ by *n* rounds of division. By 10 rounds of division, the cell number increases by 10^3^. According to this assumption, 30 rounds of cell division (10^9^ ≈ 2^30^) are required for the number of cells to become 10^9^ and 40 rounds of cell division that are required for it to become 10^12^. A typical proliferating human normal cell divides on average every 24 h [27] and the cell cycle times of gastric and colon cancers have been shown to be approximately 5.4–12.3 and 4.2–7.0 days, respectively [28]. Therefore, according to these calculations, a 1-cm tumor may grow into a 10-cm tumor within 1–3 months; however, clinically, this period is too short, so that assumption is wrong. In reality, the tumor experiences substantial attrition during each cell generation. The number of successive cell generations required to generate a 10-cm tumor is large but incalculable in the absence of precise knowledge of the attrition rates [29]. Moreover, tumor heterogeneity exists. According to the “cancer stem cell theory”, all tumor cells do not possess equal growth ability and the tumorigenic tumor cells may be a rare population [30]. In recent years, tumor growth has been shown to be greatly affected by not only the tumor cells themselves, but also the surrounding microenvironment. The immune cells, capillaries, basement membrane, activated fibroblasts, and extracellular matrix (ECM) constitute the tumor stroma. Fibroblasts are a dominant component of the tumor stroma and cancer-associated fibroblasts (CAFs) play an important role in cancer progression [31]. Conversely, the high number of immune cells, such as tumor-infiltrating lymphocytes (TILs), has been shown to be associated with a favorable prognosis [32]. The influence of the tumor microenvironment may, therefore, be one of the causes of the above discrepancy.

In the clinical setting, the most important indicator is the tumor doubling time, which refers to the time it takes for the volume of the tumor mass to double. This concept was described in 1956 by Collins, who observed the size of pulmonary metastasis and Wilms’ tumors over time and found that human cancers tend to grow exponentially [33]. Many models of tumor growth, including the Gompertzian model [34], have been proposed to describe the clinical data; however, tumors appear to grow exponentially or be approximated to exponential growth in the tumors observed clinically [35]. Collins and colleagues calculated the doubling time for pulmonary metastasis from carcinomas of the colon and rectum and showed that they were distributed over a wide range. They also found that the doubling time varied for each patient, but was constant in each. These values affected the clinical onset of the primary tumor and recurrence in each patient and were related to the clinical course [36]. Spratt made a similar observation about untreated pulmonary metastases and showed that the growth rates of these tumors correlated with the survival times of the patients [37]. The tumor doubling time is calculated using the following equation:

$$ {\text{Tumor doubling time}} = \frac{t}{3} \times \frac{\log 2}{{\log d_{2} - \log d_{1} }}, $$

where *t* is the period of *d*_1_–*d*_2_, *d*_1_ is the tumor diameter measured before, and *d*_2_ is the tumor diameter measured after.

One retrospective study reported on the tumor doubling time of esophageal cancer, as measured on X-ray films. This study found that the average doubling time of 19 lesions in 18 patients was 6.7 months, although in three, it was within 1 month [38]. Gastric cancer studies utilizing X-ray film revealed similar findings, with the doubling time ranging widely from 54 to 3462 days [39–42]. In reports in which cases were classified into early and advanced stage, it ranged from 577 to 3462 days for early gastric cancer and from 105 to 305 days for advanced gastric cancer [39, 40]. Fujita argued that the development became faster as the gastric cancers progressed [43], but Takahashi et al. pointed out that gastric cancer with fast proliferation was likely to be discovered as advanced cancer [41]. Welin measured the magnitude of 375 cases of primary colorectal cancer, using the double contrast enema method, and reported that the doubling time ranged from 138 to 1155 days [44]. It was also reported that early colorectal cancer grows slowly when the cancer is limited to the mucosa, but as the tumor grows down to the submucosa, the growth speed accelerates, the doubling times for the respective stages being 31.2 vs. 25.8 months. The doubling times of the early cancers were longer than those of the advanced cancers [45]. These studies demonstrate that the tumor doubling time is dependent on the affected organ and the case and has a wide distribution ranging from days to months. When the size of the cells is 10 μm, 30 doublings are required to become 1 cm in diameter and 40 doublings are required to become 10 cm in diameter. In tumors that have a doubling time of 100 days, 1000 days (approximately 3 years) are required for the tumor diameter to increase from 1 to 10 cm.

Furthermore, it was shown that the blood concentration of tumor markers, such as alpha-fetoprotein (AFP) or carcinoembryonic antigen (CEA), increased exponentially in patients with gastric or colorectal cancers. The tumor marker doubling time was calculated to be nearly equal to the tumor doubling time obtained from images from the same patients. Takahashi concluded that the tumor marker doubling time represented the cancer growth rate and was clinically useful as a surrogate to determine the tumor doubling time [46].

In a different study, Nakamura evaluated the size of gastric cancer tumors by the surface area, rather than by the volume, according to the idea that the stomach is a planar organ. He analyzed cases of early gastric cancer in which the microscopic lesion was tracked in a retrograde direction. Based on his findings, he suggested that an approximation to the quadratic curve was possible and proposed the following function [47]:

$$ S = 0.3t^{2} - 1.1t + 3.5, $$

where *S* is the surface area (cm^2^) and *t* is the elapsed time (year).

Furthermore, he referred to the scirrhous type of gastric cancer consisting of undifferentiated cancer cells. There are only a limited number of cases of scirrhous-type gastric cancer in which annual medical examinations were performed and did not reveal abnormalities; however, within a year, the stomach begins to resemble a leather bottle. Using the aforementioned formula, he calculated the time that elapsed to completion of this disease, concluding that 6–8 years passed from the development of the cancer to completion of the typical scirrhous cancer. He also speculated that from 1 to 2.5 years were necessary for latent scirrhous cancer, such as pre-scirrhous cancer without overall curing, to transform into typical scirrhous cancer [48].

Like primary tumors, the growth rate of metastatic tumors has been studied extensively. Regarding lung metastases, it was previously reported that the mean tumor doubling time for metastasis from colorectal cancers was 109–118 days [36, 37, 49]. Moreover, the tumor doubling times for liver metastasis and gastric/colorectal cancers were observed to be 24.7 ± 11.8 and 68.2 ± 33.4 days, respectively [46]. The tumor doubling time for lung metastases was also longer than that for liver metastases and there was a tendency for the growth rate of lung metastases to be slower. The tumor doubling time of metastatic tumors was shorter than that of primary tumors. Interestingly, Takahashi found that the ratio of the growth rates of primary and metastatic tumors is constant in the same case. For instance, the ratio of the tumor doubling time for advanced gastric cancer and liver metastases is 4–5 [50].

The timing of metastasis is an interesting field of study. The diameter of clinically detectable tumors is approximately 1 cm, but how long does it take for liver metastasis from gastric cancers to become 1 cm in diameter? It was predicted that the growth rate of liver metastasis smaller than 5 mm would be faster than the growth rate of a metastasis larger than 5 mm; therefore, in some cases, half of the doubling time was applied for smaller tumors. In addition, according to the pathological examination, liver metastasis often occurred as a cluster rather than as a single cell, so it was assumed that the initial size of liver metastases was approximately 30 μm. According to this assumption, 22.2 doublings were required to become 5 mm in diameter and 25.2 doublings were required to become 1 cm in diameter. When the tumor doubling time is 24.7 days, the time required to become 1 cm in diameter is 622.4 days (1 year and 9 months) if the tumor doubling time is stable, and 348.3 days (1 year) if it is reduced by half due to an initial small size. It was reported that the median interval between gastric and hepatic resection in patients with metachronous metastases was 20 months and the metastases which relapsed within 1 year may have existed before surgery.

## Clinical application

How is the chronology of cancer clinically relevant? Although it is known that removing adenomatous polyps prevents colorectal cancers, the optimal surveillance interval of total colonoscopy has not yet been established. A previous report on the chronology of early colorectal cancers analyzed 16 lesions of depressed-type early colorectal cancers generated after the removal of all detected polyps. The mean growth rate of the depressed colorectal cancers was estimated to be 0.0406 mm/day. When the submucosal invasion was assumed to occur at an average rate of 15 mm, the average period that colorectal cancers remained in the mucosa was 370 days (12.2 months). According to this calculation, the author concluded that 1 year is the optimal interval of surveillance [11].

Another report calculated the interval of postoperative surveillance after radical surgery according to the tumor growth rate. When the maximum detectable tumor diameter using a CT scan was assumed to be 5 mm and the clinically expected size was up to 2 cm, the appropriate interval of postoperative surveillance was determined to be the time necessary for the tumor diameter to increase from 5 mm to 2 cm. This interval was calculated to be 90 days if the mean tumor doubling time was 15 and 300 days if the mean tumor doubling time was 50 days. These authors recommended that the appropriate interval of surveillance was 3 months to within 1 year after surgery [12].

It is well known that the tumor shrinkage effect of chemotherapy does not necessarily reflect the survival of patients with gastrointestinal cancers. The calculation of the natural history, using the tumor size or the level of tumor markers, has been proposed as an alternative indicator to obtain the survival benefit achieved by anticancer agents [13].

Finally, the Precision Medicine Initiative announced in the State of the Union address by Barack Obama, the former President of the United States of America, in January, 2015, is receiving worldwide attention. Precision medicine categorizes patients into carefully defined patient groups by taking into consideration individual differences, such as genome, environment, and life style, and then establishes optimal medical care for each category. Currently, a detailed cancer genome analysis is performed using a next generation sequencer (NGS), making it possible to detect the comprehensive somatic mutations that exist in cancer cells. To establish clinically useful categories based on these data, it is important to gather various patient data. It is thought that the parameters using time axes, such as the tumor growth rate, will be useful for achieving this objective. As a result, the chronology of cancer may still be useful in the era of Precision Medicine [51].

## Conclusions

To develop a novel therapeutic strategy for cancer, the responsibility often falls on the clinician to clarify the biological characteristics of the cancer. The histopathological findings are one of the major indicators of the characteristics of gastrointestinal cancer, as is the tumor growth rate measured quantitatively during the chronology of cancer. Many essential problems, such as the stagnant tumor growth rate, remain; however, even without these problems, it may be more insightful to look at the chronology of cancer in addition to the molecular biology.
